# Multiple Mechanisms of Dopamine Modulation of Neuronal Excitability and Neurotransmission in the Striatum: A Personal and Historical Perspective

**DOI:** 10.3390/cells15181627

**Published:** 2026-09-08

**Authors:** Carlos Cepeda

**Affiliations:** IDDRC, Jane and Terry Semel Institute for Neuroscience & Human Behavior, David Geffen School of Medicine at University of California Los Angeles, Los Angeles, CA 90024-1759, USA; ccepeda@mednet.ucla.edu

**Keywords:** dopamine, glutamate, NMDA receptors, electrophysiology, modulatory effects, neurotransmission, presynaptic and postsynaptic mechanisms, heteromers, oligomers

## Abstract

3,4-dihydroxyphenethylamine, commonly known as dopamine (DA), is a neuromodulator that fine-tunes neuronal excitability, neurotransmitter release, and the effects of other neurotransmitters on postsynaptic neurons. DA, acting on D1 and D2 receptor families, is involved in myriad functions. In the striatum, it is mainly implicated in motor control, motivation, and reward mechanisms. In the cerebral cortex it participates in attention processes, working memory, long-term memory, etc. DA overproduction or deficits lead to neuronal circuit imbalance that underlies a number of neurological and psychiatric diseases, including Parkinson’s disease (PD), schizophrenia, Huntington’s disease (HD), and substance use disorders (SUDs), to name a few. DA regulates neuronal excitability by modulating ion channels, the release of excitatory (glutamate) and inhibitory (γ-aminobutyric acid, GABA) neurotransmitters, and postsynaptic interactions with glutamate and GABA receptors. Together, these pre- and postsynaptic actions of DA underlie a number of synergistic or antagonistic actions that have important implications for setting membrane potentials, improving the signal-to-noise ratio, and directing the sign of synaptic plasticity. In this review, I will first provide a historical overview of the many studies exploring DA actions in the brain, with particular focus on the striatum. Then, I will emphasize some of the contributions of our laboratory to the understanding of DA modulatory effects from an electrophysiological perspective. Finally, I will discuss the mechanistic and therapeutic implications of DA function and dysfunction.

## 1. Introduction

Dopamine (DA) modulation of neuronal excitability, neurotransmitter release, and postsynaptic responses has been a fascinating subject for the past 60 years. In particular, DA interactions with ionotropic and metabotropic receptors have received special attention due to their relevance for synaptic plasticity, memory, attention, as well as for neurological and psychiatric disorders including Parkinson’s disease (PD), Huntington’s disease (HD), schizophrenia, major depressive disorder (MDD), substance use disorder (SUD), etc. D1 (D1 and D5) and D2 (D2, D3, and D4) DA receptor families are abundant in cortical and subcortical areas and through opposite or complementary actions they help maintain brain homeostasis. Too low or too high levels of DA disrupt neuronal circuits, leading to a wide spectrum of pathological conditions. DA effects are mediated by both pre- and postsynaptic DA receptors to produce a wide variety of outcomes. The focus of this personal and historical review is on the different ways that DA modulates neurotransmitter release and postsynaptic excitability. It goes without saying that, considering the extensive nature of the field, it would be practically impossible to include and do justice to the numerous neuroscientists that have contributed to the understanding of DA actions in the brain. Also, because this review is a personal account, it will be biased towards mostly electrophysiological and pharmacological effects of DA and DA-glutamate receptor interactions in the striatum. For these shortcomings I apologize.

## 2. Historical Overview

Studies on DA modulation of neuronal excitability in mammalian brains started in earnest in the mid-1960s and early 1970s in anesthetized cats and rats. Numerous studies were published using extracellular recordings with sharp electrodes and microiontophoresis for drug applications (for methodological details and a summary of these studies I refer the reader to Supplementary Figure S1A and Appendix A. The pressing question neurophysiologists attempted to answer was whether DA exerted excitatory or inhibitory actions on striatal neurons [1]. Except for sporadic observations of excitatory responses, the initial consensus was that DA was predominantly an inhibitory neurotransmitter [2,3,4,5,6,7,8]. However, electrical stimulation of the substantia nigra to release DA found both depressant and facilitatory responses from caudate nucleus neurons [9]. Interestingly, intracellular recordings also described a complex picture of DA effects on caudate neurons. Thus, stimulation of the nigro-striatal pathway resulted primarily in depolarizing, excitatory responses in medium-sized spiny neurons (MSNs) [10] or a mix of excitatory and inhibitory postsynaptic potential (EPSP-IPSP) sequences [11]. Nonetheless, in terms of the relative magnitude of the excitatory response, when substantia nigra, thalamic, and cortical inputs were stimulated individually, the cortical excitation always predominated over the other two stimulation sites [12]. Further, these authors postulated that the IPSP was the result of intrinsic GABAergic MSN collateral inhibition. The idea that DA is the excitatory neurotransmitter of the nigro-caudate pathway [10,13] was in direct opposition of later studies demonstrating that microiontophoretic application of DA to caudate or cortical pyramidal neurons (CPNs) consistently depolarizes the membrane but simultaneously inhibits spontaneous or glutamate-induced firing and the fact that DA did not modify action potential morphology or membrane input resistance argued against the idea that such inhibition was caused by Na^+^ depolarization block [14,15]. In other studies, predominant depolarizing effects also were observed after prolonged microiontophoresis of DA onto caudate neurons, along with reduced firing rates that, again, were not caused by Na^+^ depolarization block [16]. The mixed effects were attributed to DA hyperpolarization of the axon hillock, whereas the slow depolarization was attributed to activation of the dendritic region.

By the late 70s, the predominant idea was that, although caudate neurons respond to iontophoresis of DA predominantly with inhibition, their response to electrical stimulation of the substantia nigra can be either inhibitory, excitatory, or mixed. The most parsimonious conclusion at the time was that the function of DA is not so much to excite or inhibit caudate neurons as it is to modulate the response to other excitatory or inhibitory inputs [1]. DA effects also varied depending on the dose applied (i.e., low vs. high iontophoretic current intensities). Thus, at low DA concentrations glutamate-induced excitation was enhanced. In contrast, when DA was applied at high doses, glutamate excitation was reduced. The authors concluded that DA acts to modulate the efficacy of other neurotransmitters impinging upon striatal neurons [17]. In support of this hypothesis, when a D1 agonist was applied, it potentiated the activation produced by glutamate at low ejection currents, whereas at higher ejection currents, it depressed the majority of the caudate neurons. By contrast, a D2 receptor agonist was less effective at modifying their firing rate [18]. From these studies in vivo, one could conclude that DA generally inhibits spontaneous or glutamate-induced firing, provided high ejection currents are applied.

Further insights into DA modulation mechanisms were gathered using slices and intracellular recordings of striatal neurons in combination with pharmacological application of drugs under controlled conditions (Supplementary Figure S1B). The in vitro slice preparation was exploited extensively by several groups (to name a few, Giorgio Bernardi in Rome, Michael S. Levine in Los Angeles, Jose Bargas and Elvira Galarraga in Mexico, James D. Surmeier in Chicago; Shuji Takaori in Kyoto, and others). Congruent with in vivo studies, one consistent finding was that DA decreases the excitability of striatal MSNs and it was suggested this effect occurred via pre- and postsynaptic mechanisms without affecting cell membrane input resistance [19]. As to which DA receptor subtype mediated inhibitory effects, bath application of micromolar concentrations of a selective D1 receptor agonist mimicked DA effects, and this inhibition occurred by reducing a voltage-dependent Na^+^ conductance [20]. Importantly, and also in support of some in vivo studies, D2 receptor agonists had no detectable effects. Also, similar to some in vivo studies, low concentrations of bath applied DA (1 µM) produced membrane depolarization and increased firing, whereas high concentrations (100–500 µM) inhibited spontaneous action potentials without apparent changes in membrane potential [21]. These authors suggested that D1 receptors mediated the inhibitory effect and D2 receptors mediated the excitatory effect [21,22].

However, D1 excitatory effects also were encountered. In a landmark study [23], the authors found a way to reconcile contrasting effects demonstrating that the response of MSNs to D1 receptor agonists vary depending on their membrane potential. Discharges evoked by depolarizing current pulses were inhibited by D1 receptor agonists at very hyperpolarized membrane potentials, but at depolarized membrane potentials D1 agonists enhanced evoked activity. The potentiating effects were mediated through facilitation of L-type Ca^2+^ currents. Similarly, another study found that incubation of slices with a D1 receptor agonist resulted in dose-dependent potentiation of EPSCs in MSNs [24].

The discovery that the D1 and D2 dopamine receptors are segregated in direct and indirect pathway MSNs [25,26] shed new light on DA modulatory mechanisms and provided a potential mechanism for the contrasting effects of DA. Although the discovery of DA receptor segregation was initially challenged [27,28], and there is some evidence that a small percentage of MSNs (~6% in dorsal striatum) contain both receptor subtypes (for a review, see [29]), the prevailing consensus favors DA receptor segregation in direct and indirect pathway MSNs.

Thus, the current view is that DA effects on neuronal excitability as well as on glutamate-induced firing can be inhibitory or excitatory depending on several factors, including the DA receptor subtype present in MSNs, the level of membrane polarization, and the dose of DA applied. However, the inhibitory effects may not be due solely to DA release from substantia nigra terminals. Studies on neurotransmitter co-release added another layer of complexity but, at the same time, helped understand DA actions. Indeed, there is growing evidence that DA neurons in the nigrostriatal pathway co-release GABA [30,31], which could explain some of the inhibitory effects of DA.

In the next sections, I will present a more detailed examination of pre- and postsynaptic mechanisms of DA modulation. A word of caution is appropriate here as I need to point out that, in mammalian brains, the dichotomy of pre- vs. postsynaptic mechanisms is artificial since an absolute distinction of both mechanisms is practically impossible. Nonetheless, for the sake of simplicity, I have adopted this somewhat artificial separation.

## 3. Predominantly Presynaptic Modulation of Glutamate Release in Striatal Neurons

In primitive organisms, such as crustaceans or Drosophila melanogaster, DA has been shown to modulate responses at the neuromuscular junction by presynaptic mechanisms. For example, in the prawn, DA produced a concentration-dependent decrease in the size of the excitatory junctional potential without affecting muscle fiber resting membrane potential or input resistance [32]. Because it did not affect the amplitude distribution or decay time of quantal unit currents, the authors proposed a presynaptic mechanism of action. Further, DA reduction in neurotransmitter release was suggested to be due to a decrease in the entry of Ca^2+^ during the nerve terminal action potential. Similarly, in Drosophila, application of DA on neuromuscular preparations resulted in a dose-response decrease in the excitatory junction potentials. In addition, the quantal evoked transmission showed a depression of vesicular release and, because DA did not alter the shape of the spontaneous synaptic currents, the authors also suggested a presynaptic site of action [33].

Several receptor binding and immunohistochemical studies hinted at the possibility of the existence of DA D2 receptors on corticostriatal terminals [34,35,36,37,38]. However, findings were not always unanimous [39,40,41]. In contrast, pharmacological studies were more consistent in supporting the existence of presynaptic D2 receptors capable of regulating glutamate release [42,43,44,45,46,47].

Electrophysiological studies demonstrating pre- vs. postsynaptic actions of D2 family receptors also have been inconclusive. Studies examining the effects of DA found that both pre- and postsynaptic mechanisms were implicated in the reduction of striatal excitability [19,48]. In vitro experiments initially suggested that the reduction of excitatory postsynaptic potentials by DA was mediated exclusively by postsynaptic D1 receptors [20]. D2 receptor-mediated effects were not observed unless these receptors were rendered hypersensitive by DA-depleting lesions [49]. However, subsequent studies demonstrated that D2 receptor-mediated effects were not always dependent on rendering these receptors hypersensitive [50,51].

**Figure 1:**
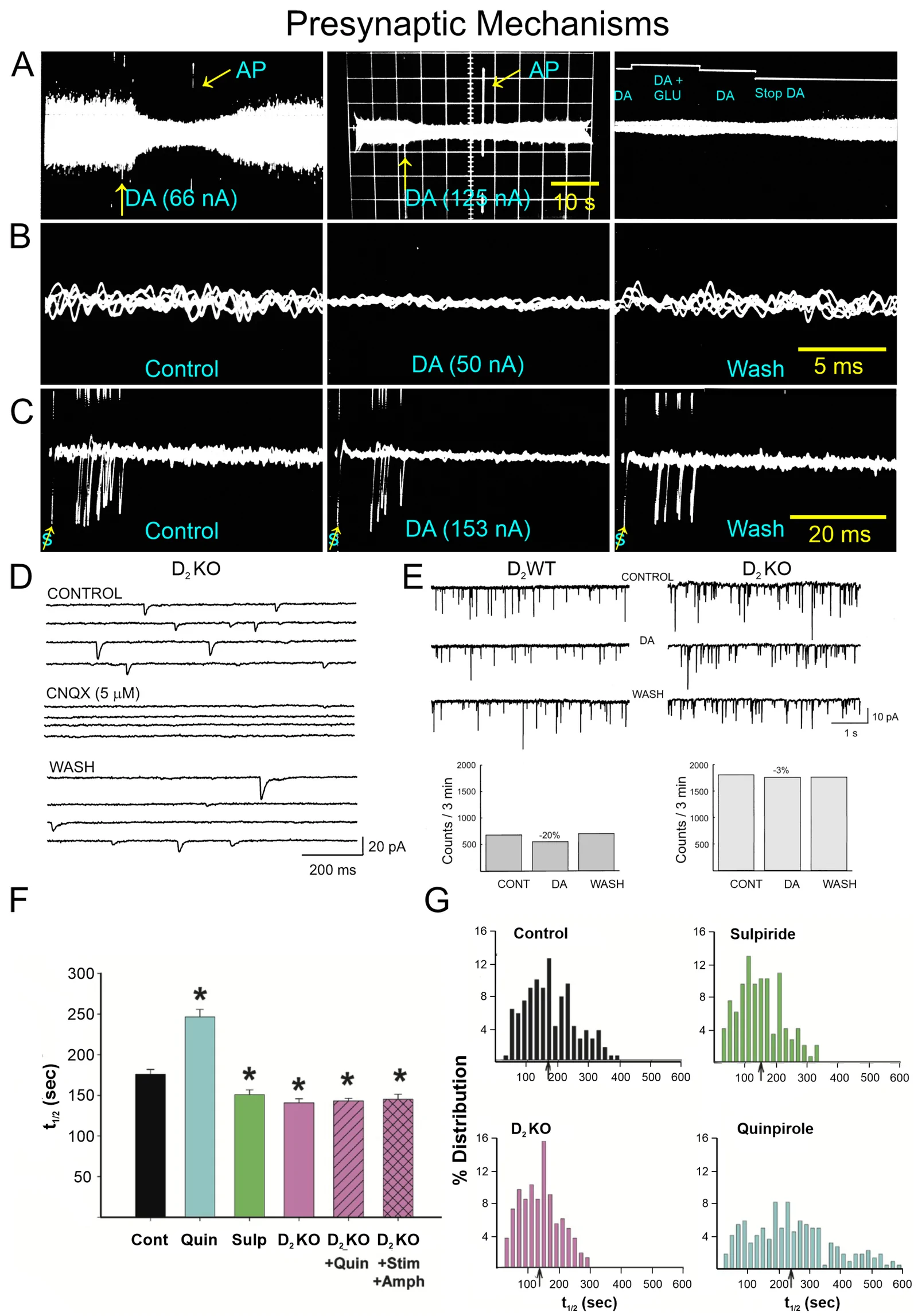
Extracellular recordings of synaptic noise and single unit activity in vivo (for a detailed explanation of the different recording techniques I refer the reader to Supplementary Figure S1). (**A**) The left panel shows the effect of DA iontophoresis in an MSN from cat striatum. During drug application, background noise was dramatically reduced. An action potential can be seen during drug application. The middle panel shows a similar effect in another MSN from rat striatum. The right panel illustrates the effect of adding glutamate (Glu) by iontophoresis in the presence of DA. After DA application alone, synaptic noise was reduced, Glu application produced an increase in noise levels, and after both DA and Glu were removed, the original noise level recovered. (**B**) Fast sweeps recorded from the cat neuron shown in A before, during and after DA iontophoresis. (**C**) In another cat neuron the frontal cortex was stimulated electrically to induce responses in the caudate. DA application reduced the number of action potentials as well as background synaptic noise, both of which recovered after DA was discontinued. (**D**) Intracellular recordings with sharp electrodes in rat slices. Continuous recordings before (control), during bath application of the AMPA receptor antagonist 6-cyano-7-nitroquinoxaline-2,3-dione (CNQX), and after (wash) to demonstrate spontaneous synaptic events were indeed mediated by AMPA receptors. Holding potential was −60 mV, and all recordings were made in the presence of 10 μM bicuculline (BIC). (**E**) Spontaneous synaptic currents recorded in neurons from a WT and a D2 receptor-deficient mutant. Traces from each cell show examples of membrane current recorded over a 5 s period before (control), during bath application of DA (20 μM), and after its wash-out (wash). Graphs show plots of the total number of events detected in each condition during a 3 min period. In the neuron from the WT, DA produced a 20% decrease in the frequency of events while in the D2 receptor-deficient mutant there was virtually no change. (**F**) Distributions of terminal half-times of destaining in the presence of the D2 receptor agonist quinpirole (Quin; 0.5 μM) and antagonist sulpiride (10 μM) were compared to controls (D2 wild-type mice). Destaining half-times in D2−/− mice were similar to those for sulpiride. D2−/− mice exposed to quinpirole (0.5 μM) or to a combination of amphetamine (10 μM) and striatal stimulation also show low half-times of destaining. * *p* < 0.05. (**G**) Histograms show distributions of t1/2 values. Arrows indicate mean values that shift to the left (more rapid destaining) with sulpiride and in D2−/− mice and shift to the right with quinpirole. Modified from [58,60].

In the early 1980s, in the course of our studies of DA modulation of MSN responses evoked by cortical stimulation we made an interesting, incidental observation that led us to hypothesize that presynaptic effects could be demonstrated electrophysiologically by studying random voltage fluctuations, i.e., background synaptic noise. In anesthetized, acute preparations of cats or rats, caudate neurons are silent due to highly hyperpolarized resting membrane potentials. As a result, background noise recorded extracellularly using high-impedance sharp microelectrodes is not generated by action potentials from neighboring neurons but more likely by random spontaneous synaptic potentials. When driving the microelectrode from the surface of the caudate, different regions are easily discernable with respect to background noise: “idle” zones, where background activity approaches the noise of the recording system and “active” zones, where the noise levels increase two- to threefold. These active zones are most of the time a secure indication of the close proximity of a neuron. With no further manipulation, background noise remains constant for long periods of time. A reliable means to confirm the close proximity of a neuron is to stimulate the corticostriatal pathway or to apply, iontophoretically, an excitatory amino acid such as glutamate. Notably, we observed that such background noise can be modulated pharmacologically. Indeed, in some MSNs, iontophoretic applications of DA close to the recorded neuron produced a significant decrease in background synaptic noise and lasted as long as DA was present (Figure 1A,B). Background noise recovered progressively until it reached preapplication levels. In a few spontaneously active neurons, DA application first produced an inhibition of action potentials and subsequently a reduction of synaptic noise. In contrast to DA, GABA iontophoresis had no observable effect on background noise. Since both drugs are ejected as cations (with positive current), the possibility of a current artifact could be dismissed. Regardless, current controls also were performed and in no single case Na^+^ iontophoresis reproduced DA effects. In other experiments, we also stimulated the motor cortex and examined the response of the caudate neuron. During DA-induced noise depression, the neuron was less responsive to cortical stimulation (Figure 1C). However, responses could be elicited again by increasing the stimulus intensity. Overall, these observations pointed to a DA modulation of neurotransmitter release via presynaptic actions.

If indeed DA modulates glutamate release from corticostriatal terminals, one could expect that DA-depleting lesions would influence glutamatergic transmission. Before the introduction of whole-cell patch clamp recording technique, observation of spontaneous, low-amplitude voltage fluctuations during intracellular recordings occurred rarely. However, several studies reported that, after DA depletion those spontaneous, random voltage fluctuations (also known as “synaptic noise”, or “spontaneous depolarizing potentials”) were more apparent [52,53]. The specific type of neurotransmitter causing spontaneous synaptic activity could not be determined precisely. Nonetheless, these studies suggested a tonic inhibitory influence of DA on the synaptic input onto striatal MSNs [52]. In a follow-up study from the same group it was reported that the frequency of 4-aminopyridine (4-AP)-enhanced glutamatergic synaptic potentials (primarily from cortical and thalamic afferents) was reduced by quinpirole (a D2 receptor agonist) in about 35% of glutamatergic inputs, with a mean frequency reduction of ~38%. The authors estimated that only about 10–15% of terminals were modulated by the D2 agonist. In addition, because the input resistance and membrane potential did not change during quinpirole application, a presynaptic site of action was suggested [54]. Similar conclusions were put forward by Bernardi’s group. Although initial observations in acute or short-term DA-depleted animals did not find increased number of striatal neurons showing spontaneous depolarizing potentials [55], long-term depletion did find a higher percentage of MSNs displaying these potentials and a D2 receptor agonist reduced their frequency, partially caused by supersensitivity of presynaptic D2 receptors [49,56]. However, it is important to remember that only a subset of terminals are modulated by D2 receptor agonists. Notably, the number of terminals modulated by quinpirole agree with previous anatomical observations showing that corticostriatal boutons containing D2 receptors represent a minority [35,36,57], although this could be an underestimate.

The advent of the whole-cell patch clamp technique allowed the recording of spontaneous synaptic currents at much better resolution than traditional intracellular recordings with high-impedance sharp electrodes. With this technique, it was demonstrated that, in fact, all MSNs display spontaneous glutamatergic and GABAergic activity depending on the voltage membrane potential. Further support of presynaptic effects of DA on glutamate release was provided by studies in D2 receptor knock-out (KO) mice. In D2 receptor-deficient mutants, there was an increase in the frequency of spontaneous glutamate receptor-mediated inward currents without a change in mean amplitude. Bath application of 4-AP and bicuculline significantly increased the frequency of spontaneous depolarizing potentials in both D2 receptor-deficient mutants and wildtype (WT) mice. However, D2 receptor KO mice displayed significantly more excitatory synaptic events than control mice [58] (Figure 1D,E). Further, in WT mice, activation of D2 family receptors with DA or quinpirole decreased spontaneous excitatory events and conversely sulpiride, a D2 receptor antagonist, increased activity. In D2 receptor-deficient mice, sulpiride had no or very little net effect. We concluded that D2 receptors at presynaptic glutamatergic terminals could function as gatekeepers of glutamate release and thus may protect striatal neurons from excessive excitation [58]. Interestingly, while high-frequency stimulation of corticostriatal fibers induced a form of long-term depression (LTD) in WT mice, D2 receptor KO mice showed N-methyl-D-aspartate (NMDA) receptor-dependent long-term potentiation (LTP) [59].

Using alternative splicing of the D2 receptor gene, at least two isoforms have been identified, D2L (long) and D2S (short). In the brain, the D2L form is the most prevalent [61]. Thus, the question was asked as to which one of the two isoforms could underlie presynaptic modulation of glutamate release. In an elegant study, it was documented that D2-receptor-dependent modulation of glutamate release preferentially involves the D2S receptor isoform [62].

In spite of the bulk of evidence indicating the presence of functional D2 receptors on presynaptic glutamatergic terminals, solid proof of their existence was still indirect. Definitive proof was finally provided by a technique that allows visualization of corticostriatal terminals using a fluorescent dye (FM1-43) (Figure 1F,G and Figure S1C). By combining optical monitoring of synaptic vesicle exocytosis and electrochemical detection of striatal DA release, the authors found that DA, via D2 receptors, inhibited the activity of subsets of corticostriatal terminals. Further, this regulation was dependent on the frequency of stimulation applied to these afferents. Thus, the most active inputs were selected by filtering out the activity of the less active inputs. This could be the mechanism whereby DA release associated with salience interacts with a highly activated cortical input during motor learning to reinforce specific subsets of corticostriatal connections [60]. In addition, D2 receptor-dependent reduction of glutamatergic synaptic transmission also was specifically demonstrated in indirect pathway MSNs [63]. Another irrefutable proof of DA presynaptic actions via D2 receptors under more controlled conditions was recently provided using ventral tegmental area (VTA)–cortical co-cultures [64]. The authors elegantly demonstrated that acute depletion of DA increased the frequency of spontaneous glutamate release, driven by a surge in basal presynaptic Ca^2+^ caused by D2-Gβγ-mediated disinhibition of voltage-gated Ca^2+^ channels.

## 4. Predominantly Postsynaptic Modulation of Glutamate Transmission by DA and Its Agonists

DA not only modulates glutamate release from excitatory inputs but also DA-glutamate receptor interactions at the postsynaptic level. The structural basis of DA-glutamate receptor interactions at the postsynaptic compartment of CPNs and MSNs is the “synaptic triad”, where DA and glutamatergic terminals converge onto dendritic spines [65]. As mentioned above, at low DA concentrations glutamate excitation is enhanced, whereas at high doses, glutamate excitation is reduced [17]. In addition, DA appears to affect spontaneous and glutamate-induced firing differentially. Thus, at low concentrations, DA primarily inhibits spontaneously active MSNs but is less effective at modifying glutamate excitations and produces mixed effects. When DA is ejected at high currents it tends to decrease the glutamate response. Overall, at low DA ejection currents, by reducing spontaneous activity, the net effect of DA is an enhancement of the glutamate-induced response [66]. It was thus postulated that one of the main functions of DA is to increase the signal-to-noise ratio [67].

**Figure 2:**
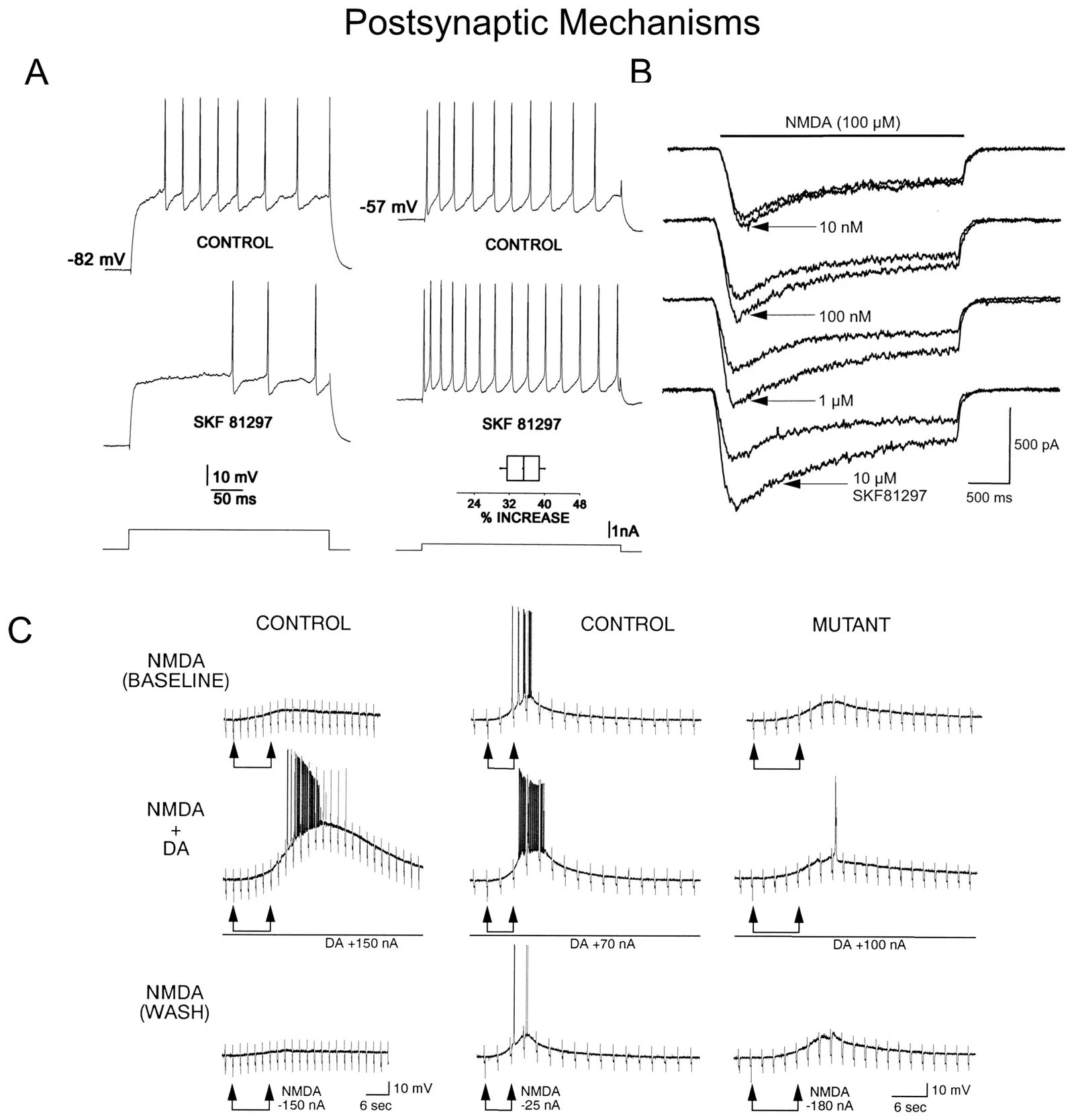
(**A**) Actions of D1 receptor agonists are inhibitory or excitatory, depending on the membrane potential. The firing response to a step depolarization at a resting membrane potential of approximately −82 mV (top, 7 action potentials) is reduced in the presence of 1 μM SKF 81297 (3 action potentials). Stimulus current is at the bottom. Stimulus and membrane potential are maintained for both conditions. In the same neuron the firing evoked from a membrane potential of approximately −57 mV (top, 10 action potentials) is increased by 1 μM SKF 81297 (middle, 14 action potentials). Stimulus at the bottom was maintained constant. Box plot shows mean firing increase for a sample of neurons in the presence of 1 μM SKF 81297. (**B**) NMDA currents are potentiated by application of the full D1 dopamine (DA) receptor agonist SKF 81297 in a concentration-dependent manner in neurons from rats. Examples of potentiation of NMDA (100 μM) current by four concentrations (10 and 100 nM and 1 and 10 μM) of SKF 81297 in a single neuron. Each pair of responses represent the control response and then the response in the presence of the D1 agonist at each concentration (←). (**C**) Example of effects of DA on responses induced by iontophoretic application of NMDA in striatal slices. Left and middle columns show cells from controls. Top traces are baseline responses to NMDA alone. Middle traces show the potentiation of the responses in the presence of iontophoretic application of DA. In all columns, DA was applied for 1 min before and during NMDA. Bottom traces show wash 4 min after DA application ceased. Right column shows a cell from a mutant. Top trace shows a depolarizing response to NMDA alone. Middle trace shows that this response was minimally altered in the presence of DA. Bottom trace shows wash 4 min after application of DA. Modified from [23,70,72] (Copyright Society for Neuroscience).

While some of the contrasting effects of DA could be partially explained by different DA concentrations or by the existence of two types of DA receptor families, it came as a surprise that DA effects could also depend on the ionotropic glutamate receptor subtype being activated [68,69]. Thirty-three years ago, we discovered that DA modulates glutamate receptor-evoked responses differentially. Thus, while responses mediated by activation of α-amino-3-hydroxy-5-methyl-4-isoxazolepropionic acid (AMPA) receptors were decreased, responses mediated by NMDA receptors were enhanced [68]. When selective DA receptor agonists were used, we observed that, for the most part, activation of D1 receptors potentiated glutamate responses, in particular those mediated by NMDA receptors, whereas activation of D2 receptors attenuated glutamate responses, in particular those mediated by AMPA receptors [68,70,71] (Figure 2C). Additional studies in our laboratory demonstrated the important role that the cAMP-PKA-DARPP-32 cascade played in the induction of differential effects by DA on striatal MSNs [72] (Figure 2B).

Although the discovery of differential modulation of glutamate responses by DA was met with some resistance, the overwhelming evidence has been in favor of our paradigm. In the cerebral cortex [73,74,75,76,77] and nucleus accumbens (NAc) [78], the majority of studies replicated our findings. The dissident voices could be divided into two groups: those that claimed that DA does not affect glutamate transmission [79,80] and those that, while accepting the observation, claimed that the underlying mechanisms are indirect, e.g., via modulation of voltage-gated channels, and have little to do with direct glutamate receptor modulation [81]. In [79] the authors observed no modulation of NMDA or AMPA responses by DA. The authors acknowledged that different modes of drug application (bath application vs. iontophoresis) could have explained the differences. More importantly, we suggested that it was possible that holding the membrane at very hyperpolarized levels could have prevented modulation. The other study showed modulation of synaptic responses by DA in nucleus accumbens (NAc) but not in dorsolateral striatum [80]. However, this study also had major procedural differences including the young age of their animals, slice conditions, and holding potential. Interestingly, it has been reported that, in very young animals, D1 receptor stimulation reduces NMDA responses in NAc but in adult animals it potentiates these responses [82]. Stimulation within the striatum and not in the cortex is another important difference, as this stimulation will induce DA release and occlude effects of extrinsic DA. Another major limitation in both studies was that selective D1 and D2 receptor agonists were not used.

While modulation can occur even when voltage-gated currents are blocked [72], DA also has been shown to modulate Na^+^, and K^+^ channel activity in dorsolateral and ventral striatal MSNs [83,84]. Whole-cell patch clamp studies in dissociated MSNs demonstrated that DA and D1 receptor agonists decrease the slowly inactivating component of the K^+^ current but do not seem to affect the rapidly inactivating component. In contrast, the application of the D2 agonist quinpirole enhanced this current [83]. Both agonists also modulated the Na^+^ current, producing significant reductions. In our studies, and those of other groups, we also showed that voltage-gated Ca^2+^ currents play an important role in the enhancement of NMDA currents [75,85]. This observation brings us back to the demonstration that some effects of DA are state-dependent, i.e., the outcome of DA modulation is dependent on the membrane potential of the postsynaptic neuron, as well as the ionic channel subjected to DA application [23,86,87]. Indeed, discharges evoked by depolarizing current pulses were inhibited by D1 agonists when holding at negative membrane potentials but at more depolarized potentials D1 agonists enhanced evoked activity [23] (Figure 2A). This effect was shown to be dependent on activation of L-type Ca^2+^ currents. Complementing this paradigm, D2 receptor activation in MSNs decreased cellular excitability by blocking L-type Ca^2+^ channels [88]. Remarkably, this paradigm is similar to the one we postulated to explain differential effects of DA on glutamate-induced responses [68,85,89]. It is important to remember that prolonged application of DA itself can depolarize the membrane, thereby removing the Mg^2+^ block and allowing Ca^2+^ channel opening.

Further insights into DA modulation of corticostriatal responses were provided by studies examining the effects of DA on direct and indirect pathways. Based on the premise that indirect pathway MSNs are more excitable and are more tightly coupled to cortical activity than direct pathway MSNs [90,91,92,93,94], it was demonstrated that D1 receptor agonists enhance corticostriatal responses in direct pathway MSNs, whereas D2 receptor agonists reduce orthodromic and autoregenerative responses in indirect pathway MSNs, thus balancing the excitability of both pathways [95]. We also used mice expressing green fluorescent protein in D1 and D2 receptor-containing cells to examine DA receptor modulation of MSNs of the direct and indirect pathways [91,96]. As expected, D1 enhancement of glutamate responses was specific to direct pathway neurons, whereas D2 attenuation of responses was specific to indirect pathway MSNs [91,96]. For this modulation to occur, particularly the enhancement of glutamate release by D1 agonists, endocannabinoids also play an important role.

With the generation of mice with specific deletion of NMDA receptor subunits, it was possible to demonstrate that the enhancement of NMDA responses by D1 agonists is significantly reduced in mice with genetic knock-down of GluN1 subunits. In addition, whereas genetic deletion or pharmacological blockade of GluN2A subunits enhanced D1 potentiation of NMDA responses, blockade of GluN2B subunits reduced this potentiation, indicating that these regulatory subunits, primarily contained in extrasynaptic NMDA receptors, counterbalance their respective functions [97]. Finally, in a series of elegant experiments combining patch-clamp recordings, Ca^2+^ imaging, optogenetics and glutamate uncaging, the investigators corroborated one of the main tenets of our paradigm, namely, that D2 receptors decrease NMDA-induced responses [63].

**Figure 3:**
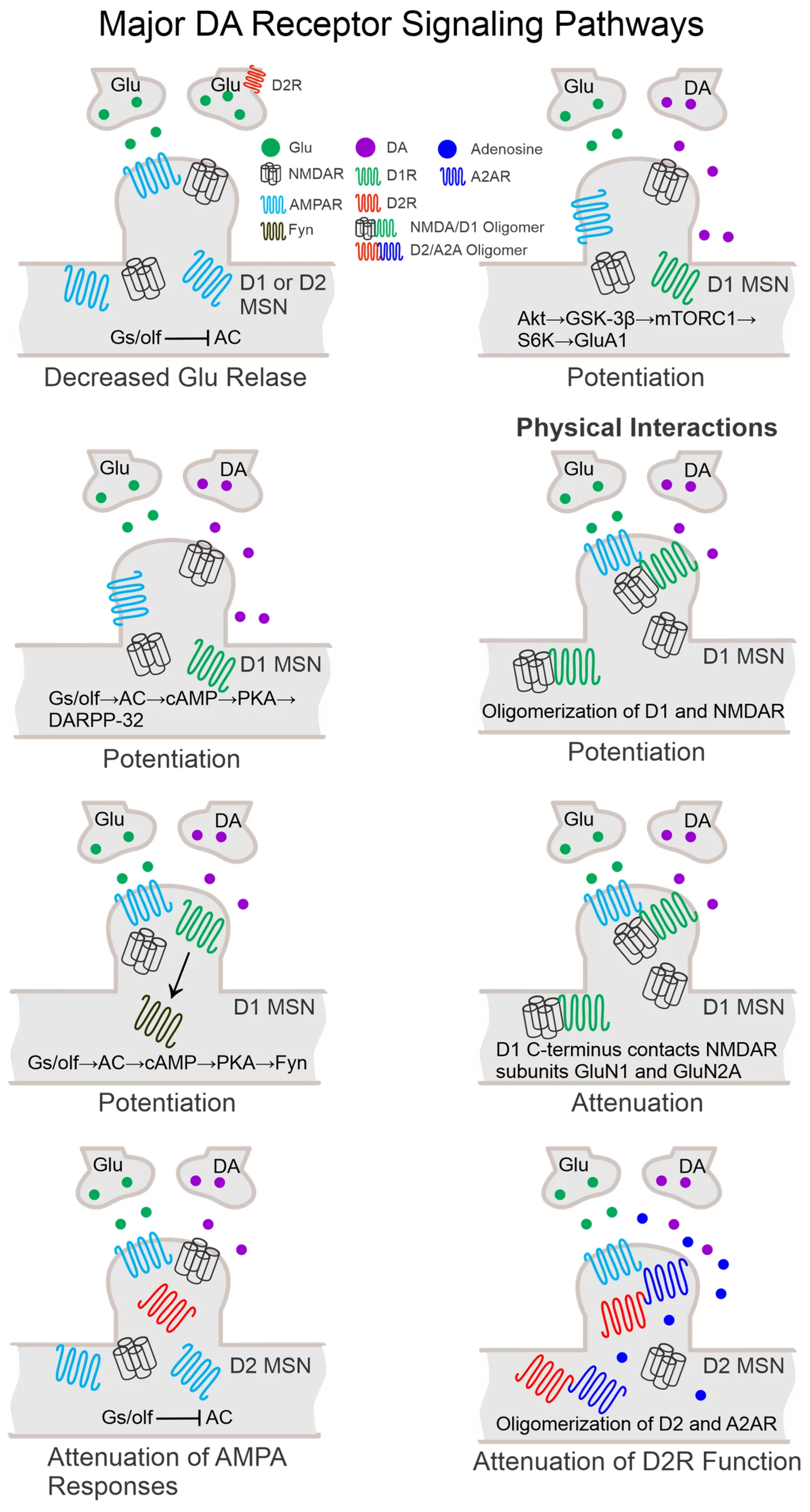
Main DA receptor signaling pathways and modulation. Stimulation of D1 or D2 receptors and glutamate receptors leads to reciprocal functional interactions through both activation of cell signaling pathways or direct physical interactions. (**Left**) DA, via presynaptic D2 receptors on cortical terminals decreases glutamate (Glu) release. DA, via D1 receptors, potentiates NMDA responses through the canonical Gs/olf-AC-cAMP-PKA-DARPP-32 pathway or by activation of Fyn. Activation of D2 postsynaptic receptors attenuates AMPA responses by inhibiting adenylate cyclase (AC). (**Right**) D1 receptor activation potentiates AMPA receptor function via the mTORC1 pathway. Physical Interactions: Oligomerization of D1 and NMDA receptor (or GluN1 subunits) potentiates NMDA responses. A physical association of D1 C-terminus with NMDA receptor subunits can attenuate NMDA responses provided the PKA and PKC cascades are blocked. Oligomerization of D2 and A2A receptors attenuates D2 receptor function.

The mechanisms involved in D1 receptor enhancement of NMDA responses are varied and complex. Intracellular signaling cascades, particularly the cAMP-PKA-DARPP-32 cascade, play a critical role [72,98,99] (Figure 3). Further, activation of D1 receptors enhances surface expression of NMDA and AMPA receptors [100,101], as well as GluN1, GluN2A, and GluN2B proteins in the synaptosomal membrane fraction [102,103], an effect that is dependent on Fyn protein tyrosine kinase [102,104]. Also, main contributors include phosphorylation of NMDA receptor GluN1 subunits [105] and activation of voltage-gated Ca^2+^ channels, particularly L-type channels [74,85]. In the NAc, NMDA receptor potentiation by phospholipase C-coupled D1-like receptors occurs via protein kinase C (PKC) activation [78]. In the cerebral cortex, the enhancement of NMDA responses by D1 agonists seems to occur via suppression of Ca^2+^/calmodulin-dependent inactivation of NMDA channels [75], although a role for the PKA cascade also has been demonstrated [106].

Evidence also suggests that D1 and NMDA receptors or their subunits can have direct physical interactions. They can be assembled as oligomeric units in the endoplasmic reticulum and then transported to the cell surface as a preformed heteromeric complex [107,108,109]. Likewise, activation of NMDA receptors recruits D1 receptors from the interior of the cell to the plasma membrane, particularly in dendritic spines, while having no effect on the distribution of D2 receptors [110]. These physical interactions can attenuate or potentiate receptor function [111].

It must be acknowledged that homomeric and heteromeric complexes are not limited to DA and NMDA receptors. It is widely known that D2 receptors form heteromeric complexes with A2A receptors and they are found primarily in indirect pathway MSNs [112] and in astrocytes [113]. As for the possible function of D2-A2A receptor complexes, one could be to limit excessive activation of D2 receptors, as each one taps on opposing signaling pathways via Gs/olf and Gi/o proteins respectively [114]. Both neuronal and astrocytic D2-A2A complexes are relevant to understand a number of disease mechanisms and drugs targeting these complexes and being sought actively [115,116].

## 5. DA Modulation of GABA Neurotransmission and Reciprocal Interactions

Although the primary goal of the present review is to examine DA-glutamate receptor interactions, a few words on the effects of DA on GABA-receptor-mediated responses are warranted, considering the importance of maintaining the excitatory–inhibitory balance in cortex and striatum. Similar to DA–glutamate interactions, much of the initial knowledge of DA-GABA neurotransmitter interactions was obtained using biochemical methods including microdialysis [117,118,119,120]. Also similar, the effects of DA are differential depending on the DA receptor subtype activated. Thus, in striatum, D1 receptors stimulate depolarization-evoked GABA release, whereas D2 receptors inhibit that release [121,122,123]. In frontal CPNs, D1 agonists can enhance sIPSCs by increasing the excitability of GABA interneurons, whereas D2 agonists produce a significant reduction in miniature (m)IPSCs and postsynaptic GABA responses [124,125]. Further studies showed that application of a D4 receptor agonist, but not by a D2 agonist, produced a decrease in GABA currents by modulating the PKA/PPI signaling cascade [126]. In the VTA, DA, via D2 receptors and inwardly rectifying potassium channels, inhibits GABA_A_ receptor-mediated IPSCs recorded from DA neurons [127]. In the external globus pallidus (GPe), the main output region of GABAergic terminals from indirect pathway MSNs, it was recently demonstrated that DA, via D2 receptors, exerts presynaptic inhibition in dorsolateral and ventromedial GPe subregions, whereas in dorsomedial and ventrolateral subregions D4 receptors induce postsynaptic inhibition [128]. It can be said that, with few exceptions, differential DA-GABA interactions fall into the same paradigm as DA-glutamate interactions.

GABAergic MSNs are interconnected through axon collaterals and DA plays an important role in lateral inhibition. Studies in slices showed that the GABA currents among MSNs are regulated presynaptically so that D1 receptor agonists facilitate whereas D2 receptor agonists depress this synaptic current [129]. Overall, these results are consistent with the demonstration that DA exerts excitatory effects on striatal interneurons via D1 receptors and inhibitory effects via D2 receptors [130].

DA can also directly affect GABA_A_ receptor-mediated responses postsynaptically. In dissociated MSNs, it was demonstrated that DA, via D1 receptors, reduces postsynaptic GABA currents by activating a PKA/DARPP-32/protein phosphatase 1 (PP1) signaling cascade that targets GABA_A_ receptor β1 subunits [131]. To further elucidate the respective role of the two D1 receptor family subtypes, in our lab, we examined the effects of DA on GABA currents in normal mice and in mice lacking the D1 or the D5 receptor subtype. Whole-cell patch clamp recordings of MSNs in slices showed that addition of a D1 agonist reduced GABA currents significantly more in D5 KO compared to D1 KO mice, indicating that D1 receptors are the main D1-like receptor subtype involved in the modulation of GABA currents [132].

We must add that GABA, via GABA_A_ and GABA_B_ receptors located on presynaptic terminals, also modulates the release of DA. Using whole-cell and perforated-patch recordings from the axons of DA neurons in mouse striatum it was shown that application of GABA depolarized axons but also decreased the amplitude of axonal spikes, limited their propagation, and reduced DA release via Na^+^ channel inactivation and shunting of the spikes [133]. In addition, ambient (tonic) GABA within the striatum is also involved in the regulation of DA release [134]. By supporting GABA uptake, GABA transporters (GAT1 and GAT3) present on striatal astrocytes play an important role in the regulation of both tonic GABA and DA release [135,136].

## 6. Implications of DA–Glutamate Interactions in the Brain

DA is a versatile molecule implicated in a large number of normal functions. Indeed, DA neurons in the midbrain participate in, among others, motor control [137], attention [138], reward prediction errors [139,140], appetitive learning [141], memory consolidation [142], states-of-vigilance [143,144], etc. When DA concentrations fall below or rise above the normal levels, the brain becomes dysfunctional as manifested by neurological and psychiatric disorders such as PD, HD, schizophrenia, SUD, severe depression, anhedonia, etc. By modulating neuronal excitability, neurotransmitter release, and postsynaptic interactions with glutamate and GABA receptors, the number of processes influenced by DA increases exponentially. At the presynaptic level, DA D2 receptors regulate the release of glutamate in the corticostriatal pathway and may play a neuroprotective role and, at the postsynaptic level, DA dictates changes in intrinsic excitability by modulating ion channels, with diverse outcomes depending on the membrane potential. DA also modifies the function of other neurotransmitters, leading to the enhancement or reduction of expected effects. In the next paragraphs I will examine in more detail some of the multiple outcomes produced by DA modulation, as well as DA interactions with glutamate receptors, in particular NMDA receptors.

### 6.1. DA, Brain Development, and Synaptogenesis

By in situ hybridization it has been demonstrated that mRNA expression of D1 and D2 receptor genes precedes DA innervation, suggesting these receptors play a role in brain development [145]. In particular, D1 family receptors are expressed in the embryonic period and may play a role in sculpting the cerebral cortex [146]. DA receptors also influence GABA interneuron migration during embryonic cortical development. Thus, activation of D1 receptors promotes cell migration, whereas activation of D2 receptors decreases migration [147,148,149]. In addition, activation of D1 or D2 receptors increases dendritic arborizations and the number of spines in MSNs co-cultured with DA producing neurons or after addition of DA or DA receptor agonists in the culture [150,151]. These observations have relevance to the understanding of developmental disorders such as schizophrenia and autism [152,153].

A weak and transient interaction between NMDA and D1 receptors also appears to play a structural and functional role in the developing brain. Using multidimensional spectral single-molecule localization microscopy to monitor the interaction between NMDA and D1 receptors, transient interactions were randomly observed along the dendritic tree of hippocampal neurons. It was higher early in development, promoting the formation of NMDA-D1 receptor complexes, favoring NMDA receptor clusters and synaptogenesis in a DA-receptor-signaling-independent manner. Preventing the interaction in the neonate, but not adult, brain alters in vivo spontaneous neuronal network activity patterns in male mice [154].

### 6.2. DA-NMDA Receptor Interactions and Synaptic Plasticity

DA–glutamate receptor interactions play a critical role in synaptic plasticity in the striatum. LTD appears to be dependent on activation of both D1 and D2 receptors [155,156]. In our hands, induction of LTD by tetanic stimulation of corticostriatal terminals was not as consistent and appeared to greatly depend on recording conditions, stimulation parameters, and location within the striatum. Similar findings were reported by another lab [157,158]. The type of preparation also seems critical for the type of long-term synaptic plasticity obtained after corticostriatal pathway stimulation in striatum. In more intact preparations (i.e., in vivo) LTP, not LTD, is observed after subthreshold tetanic cortical stimulation [159,160]. Strong evidence supports the observation that activation of D1 receptors is required for the induction of LTP [161]. Indeed, NMDA receptor-dependent LTP is blocked by DA depletion and by D1 receptor antagonists but is not affected by D2 receptor antagonists. Notably, in DA-depleted slices, the ability to induce LTP can be restored by bath application of D1 receptor agonists [162]. This suggests that activation of D1 receptors effectively amplifies selected cortical information to the striatum [163].

In the hippocampus, where memory formation has been studied in great detail, late LTP has been shown to be strongly dependent on DA. According to the Synaptic Tagging and Capture model [164], weak stimulation induces only early LTP. By contrast, stronger stimulation requires protein synthesis that allows late LTP. In a rodent model of episodic memory, intrahippocampal infusion of a D1 antagonist before encoding causes impaired memory for the new reward–location association measured 24 h later but has no effect on early memory measured at 30 min [165]. These data demonstrate that D1 receptor-mediated effects do not strongly affect encoding or early memory but do affect memory at later times [166]. Further, DA also increases protein synthesis, specifically GluA1 AMPA receptor subunits, thereby enabling DA-dependent LTP [167].

### 6.3. Substance Use Disorders (SUDs)

Substances of abuse (e.g., cocaine, alcohol, opioids, and so on) engage VTA neurons by direct or indirect mechanisms, resulting in enhanced DA signaling in their projection targets, particularly the NAc. Repeated substance exposure induces persistent neuroadaptations within mesolimbic and interconnected neural circuits that contribute to drug seeking and taking, and, in some individuals and at later stages of addiction, compulsive drug use despite adverse consequences and a high risk of relapse following abstinence [168,169]. In the case of opioids, both DA and NMDA receptors play a critical role [170,171]. Specifically, the GluN2B subunit of NMDA receptors is critical for opioid addiction [172]. In the case of alcohol use disorder (AUD), a chronic relapsing disorder that compels a subject to compulsively seek alcohol [173], both in vivo and in vitro studies have shown that this substance causes dose-dependent excitation of DA neurons in the VTA by blocking a leak K^+^ channel [174]. In turn, as in other addictions, VTA activation leads to an increase in DA levels primarily in the NAc [175]. Alcohol affects both DA and NMDA receptors. On one hand, alcohol directly inhibits NMDA receptors throughout the brain [176]. On the other, to counter reduced excitation, DA, via D1 receptors, activates VTA neurons [177,178]. GABA neurotransmission also plays an important role in AUD [179]. In addition, alcohol, via µ-opioid receptors, reduces GABA inputs onto VTA neurons, further facilitating VTA DA neuron firing [180]. Enhancement of excitatory neurotransmission also occurs in the direct pathway MSNs of the NAc and this enhancement is dependent on both D1 receptors and the mTORC1 signaling pathway [181]. Indeed, it has been demonstrated that striatal D1 receptor activation is required for alcohol stimulation [182,183] and this effect is enhanced in mice with low striatal D2 receptors, a susceptibility factor for AUD [184]. Further, as with other addictions and as a recurrent theme, alcohol exposure leads to an upregulation of GluN2B subunits [185]. Indeed, a D2-GluN2B complex is augmented in morphine addiction, accompanied by extracellular signal-regulated kinase (ERK) signaling impairment. Further, a brain-penetrant interfering peptide appears to disrupt the D2-GluN2B complex, thereby decreasing addictive-like behaviors [186].

With regard to the role of D1 and NMDA physical interactions, studies from Vanhoutte’s group found that concomitant stimulation of D1 and NMDA receptors drives formation of D1/GluN1 receptor subunit complexes. In contrast, blocking D1/GluN1 association with a cell-permeable peptide prevents D1-receptor-mediated facilitation of NMDA-Ca^2+^ influx and subsequent ERK activation. Remarkably, D1/GluN1 complexes control the D1-receptor-dependent enhancement of NMDA-receptor-mediated currents and LTP in D1-receptor-expressing MSNs and, although intrastriatal delivery of the peptide does not affect acute responses to cocaine, it reduces behavioral sensitization [187]. More recently, using genetically encoded cell-type-specific nuclear Ca^2+^ probes to monitor Ca^2+^ dynamics in the nuclei of NAc MSNs, the same group reported that D1-receptor-mediated potentiation of Ca^2+^ influx through NMDA receptors also drives nuclear Ca^2+^ transients. Notably, cocaine-treated mice showed a sustained nuclear Ca^2+^ increase in D1-MSNs. Furthermore, disrupting nuclear Ca^2+^ increases in D1-MSNs, but not D2-MSNs, blocked cocaine-induced morphological changes of MSNs and gene expression and blunted cocaine rewarding effects, opening new avenues for the treatment of SUD [188]. A physical interaction between D2 receptors and the GluN2B subunit also contributes to SUD [186].

### 6.4. Schizophrenia

There is probably no other disease where DA-NMDA receptor interactions play a more prominent role than in schizophrenia [189,190]. Notwithstanding a strong genetic and developmental component of the disease, positive and negative symptoms appear to be due to very high and very low levels of DA respectively. D1 receptors in prefrontal cortex are important for working memory [191,192]. Indeed, downregulation of D1 receptors in the prefrontal cortex produces severe impairments in working memory. These deficits can be reversed by short-term co-administration of a D1 agonist [193]. Along the same line of thought, recent studies have shown that a positive allosteric modulator of D1 receptors, DPTQ, provided long-term improvements of working memory in monkeys [194]. On the other hand, blockade of NMDA receptors using receptor antagonists such as ketamine and phencyclidine also reproduces symptoms of schizophrenia [195]. Conversely, clozapine, an antipsychotic that relieves positive and negative symptoms of schizophrenia, stimulates DA and glutamate release in the prefrontal cortex, thereby potentiating NMDA receptor-mediated responses via D1 receptor activation [196,197]. Importantly, long-term administration of second-generation antipsychotics induces postsynaptic upscaling, primarily by insertion of GluA2-lacking, Ca^2+^-permeable AMPA receptors [64].

Our lab examined the effects of deletion of DA D4 receptors from the frontal cortex of mice. Consistent with the idea that D2 family receptors dampen neuronal excitability, we found that D4 KO mice displayed increased frequency of spontaneous synaptic currents and the frequency and duration of paroxysmal discharges induced by epileptogenic agents [198]. These results suggest that the D4 receptors function as an inhibitory modulator of glutamate activity in the frontal cortex. In another study, we examined the effects of DA dysfunction on CPNs in the Disc-1 schizophrenia model. We showed male-specific increases in the ratio of excitatory to inhibitory synaptic events [199], suggesting that, similar to the DA pathway, the male GABAergic system is more vulnerable to the Disc1 mutation than females.

### 6.5. Major Depressive Disorder

If the positive symptoms of schizophrenia can be explained by excess DA in some subcortical areas such as the associative striatum [200], reduced levels of DA in the frontal cortex and the reward system can lead to depressive symptoms and anhedonia [201]. Interestingly, anhedonia also is a common symptom of schizophrenia [202], as well as of major depressive disorder (MDD) [203]. As could be expected, DA and NMDA receptors also are major players in MDD [204]. While traditional therapies for MDD included drugs that increased catecholamine levels, recent advances in the treatment of depression have shifted to blocking NMDA receptors [205]. Non-competitive antagonists such as ketamine and esketamine have demonstrated efficacy and rapid relief of depressive symptoms by blockade of NMDA receptors, primarily in cortical interneurons, and activation of the mTOR pathway [206,207,208,209]. Non-pharmacological, less invasive methods to treat MDD also are available. Notably, repeated bouts of theta burst transcranial magnetic stimulation (iTMS) of the dorsolateral prefrontal cortex increase DA levels in striatum and produce rapid relief of depressive symptoms [210]. Repetitive TMS also produces therapeutic effects by activating a specific class of CPNs, the intratelencephalic (IT) type of projection neurons in dorsomedial prefrontal cortex [211].

### 6.6. Parkinson’s Disease

As for other neurodegenerative diseases, DA and NMDA receptors play a crucial role in neuronal demise. Progressive loss of DA neurons and consequent dysregulation of glutamate neurotransmission, as well as imbalance in direct and indirect MSN pathways, result in characteristic signs of PD. Many groups around the world have contributed enormously to the understanding of basic mechanisms of PD and that of L-DOPA-induced dyskinesias (LIDs), in particular Calabresi, Surmeier, Bezard, Magill, and others (see, for example, [212,213,214,215,216,217,218]). Compared to HD, our group contribution to PD has been relatively modest. In a study on the effects of lesions of DA neurons we observed an increase in corticostriatal synaptic activity as well as enhanced gap junctional communication [53]. This finding was confirmed by another study [219]. Increased gap junctional communication could enhance basal ganglia synchrony and beta oscillatory activity, known to underlie motor deficits in PD [220,221,222]. Although the origin of this activity is still unknown, the external globus pallidus (GPe) is a strong candidate [223]. Remarkably, in postmortem tissue from PD patients, connexin-36, the major neural gap junction protein, was found increased in the putamen, GPe, and GPi compared to control subjects [224]. Increased gap junction permeability could be an important factor underlying PD basal ganglia synchrony.

We also examined changes of corticostriatal synaptic transmission in some genetic animal models of PD. Although these models hardly replicate the plethora of variables involved in the pathophysiology of PD [225,226], they have provided some important insights into synaptic dysfunction [227]. In a parkin-deficient mouse, we reported behavioral and synaptic changes consistent with dysfunction of the nigrostriatal pathway. Interestingly, parkin deficiency did not alter DA neuron survival in the substantia nigra [228]. In a premanifest PD model with alpha-synuclein overexpression, we observed decreases in the frequency of spontaneous EPSCs and higher stimulation thresholds to evoke EPSCs in MSNs, suggesting a decrease in neurotransmitter release at corticostriatal synapses [229]. In the same model using in vivo microdialysis, behavioral assessments, and patch-clamp recordings, we found biphasic changes in DA alterations. At 6 months of age, mice displayed increased tonic DA concentrations in striatum, accompanied by higher activity in the open field. In contrast, at 14 months of age, DA concentrations were reduced and were accompanied by sensorimotor deficits [230].

We are not aware of studies targeting GluN1-D1 receptor heteromers in PD. However, in LID, likely caused by enhanced function of D1 and NMDA receptors, uncoupling of D1 and GluN1 receptor physical interactions using a Tat-conjugated interfering peptide alleviated dyskinetic behaviors and downregulated the phosphorylation of DARPP-32 [231]. Reinforcing the role of NMDA receptors in LID, a recent study from Surmeier’s lab reported that LID induces an upregulation of GluN2B-containing NMDA receptors specifically in indirect pathway MSNs. Furthermore, knocking down the expression of *Grin2b* mRNA in iSPNs dramatically attenuated both the development and expression of LID [232]. It is interesting to note that similar pathogenic mechanisms can be encountered in SUD, HD, and other neurological conditions [172,233,234].

New methodology has provided much needed insights into PD mechanisms and therapeutics. For example, using optogenetics to systematically evaluate the role of particular cell types in the subthalamic nucleus (STN), a target area to stop tremor using deep brain stimulation, the authors demonstrated that therapeutic benefits were primarily the result of direct, selective stimulation of afferent axons projecting from layer V of motor cortex to the STN [235]. Chemogenetics by the use of designer receptors exclusively activated by designer drugs (DREADDs) also has shed new light on PD mechanisms and potential therapies. As a case in point, selective excitation of pedunculopontine cholinergic neurons activated residual nigrostriatal DA neurons and led to motor recovery, which correlated with improved striatal DA [236]. Excitingly, there are at least seven clinical trials, all in China, using DREADDs as potential therapies in a number of neurological diseases including one in PD patients (https://cen.acs.org/biological-chemistry/biotechnology/human-trial-chemogenetic-brain-therapy/104/web/2026/08, accessed on 15 August 2026).

### 6.7. Huntington’s Disease

Our laboratory has dedicated over 30 years to trying to understand the neurophysiological basis of HD in genetic mouse models [237,238]. In the early stages of the disease, DA neurotransmission is increased, leading to hyperkinetic movements that can be alleviated by depleting DA stores (e.g., with tetrabenazine) [239]. In contrast, in the late stages, DA deficits produce hypokinesia that can be treated by increasing DA function [240]. These alterations in DA neurotransmission affect glutamate receptor modulation and could contribute to excitotoxicity and deficits in behavioral flexibility [239,241]. In consequence, targeting both DA and glutamate receptor dysfunction could be the best strategy to treat HD symptoms. We first described a progressive disconnection between glutamatergic CPNs and GABAergic MSNs [242], probably as a result of striatal neurons attempting to cope with a hyperexcitable cortex [243]. In parallel, and as an adjuvant to reduce the consequences of hyperexcitation, spontaneous GABA synaptic activity is augmented in MSNs [244]. Using optogenetics and whole-cell patch-clamp recordings we determined that both parvalbumin- and somatostatin-expressing interneurons contribute to increased GABA activity [245], However, an unintended consequence of the corticostriatal disconnection is loss of synapses and dendritic spines, which leads released glutamate to activate extrasynaptic NMDA receptors preferentially and become toxic to MSNs [233,246,247]. In parallel, early loss of D2 receptors could facilitate glutamate release, which may exacerbate glutamate toxicity in striatal MSNs. There also is evidence that increased DA and glutamate activity could be responsible for some of the early HD phenotype and restoring the balance between glutamate and DA could be helpful to treat HD symptoms [248].

In the early stages of HD, when DA levels are high, D1 receptors and MSNs of the direct pathway are overactive [249]. However, blockade of D1 receptors is not advised due to widespread, adverse side effects. Interestingly, a downstream target of D1 receptors, Fyn protein, a member of the Src kinase family [250], is reduced in HD models due to decreased phosphorylation of synaptic GluN2B-containing NMDA receptors and reduced cAMP response element-binding protein (CREB) activation, conducive to cell death. Notably, expression of an active form of Src kinase promotes CREB activation, reduces caspase-3 activation, and reestablishes synaptic NMDA receptor localization in YAC128 mouse striatal neurons [251].

Little is known about the possible formation of GluN1-D1 heteromers in HD or if targeting these complexes would be a rational end effective approach. However, some studies have found co-localization of D1 and H3 receptors in MSNs of the direct pathway [252], as well as the formation of D1 and histamine H3 receptor heteromers, which exert negative interactions by reducing cAMP accumulation [253]. In HD mouse models and human cases (grades 1–2) in the early stages, it was found that D1-H3 receptor heteromers are expressed and functional in early but not late stages. Further, an H3 antagonist showed neuroprotection and improved learning and long-term memory deficits in mouse models [254].

## 7. Future Perspectives and Outstanding Questions

After more than 60 years of intense research on DA modulation of neuronal excitability, neurotransmitter release, and receptor interactions, the ground is still both fertile and fascinating.

Since the initial question was formulated (i.e., is DA excitatory or inhibitory?), knowledge of the role of DA in the brain has increased exponentially. With the advent of new methodology to measure DA release and physical receptor interactions, the future of DA research anticipates exciting new discoveries.

One of such methodological advances is the use of genetically encoded GPCR-activation-based-DA (GRABDA) indicators, e.g., dLight1, dLight3, and GRABDA, that allow fast and accurate measurements of DA dynamics in behaving animals with subcellular resolution, millisecond kinetics, and improved molecular specificity [255,256,257]. Incidentally, glutamate sensors also exist, e.g., the intensity-based glutamate-sensing fluorescent reporter (iGluSnFR) [257,258], and it will be interesting to analyze DA–glutamate interactions using both fluorescent sensors.

Other modern techniques have been used to define different types of cells in the ventral midbrain. For example, single-cell RNA sequencing (seq) identified 25 molecularly defined human cell types, including five subtypes of radial glia-like cells and four types of DA neuron progenitors [259]. Another significant advancement has been the use of single-cell genomic profiling to characterize DA neurons in the SN pars compacta [260]. By Slide-seq, at least ten groups of DA neurons were identified in control humans and PD patients, with one population expressing specifically a gene with high susceptibility to the malady [261]. Further, single-cell spatial transcriptomic and DA neuron translatomic analysis of young and old mouse brains allowed characterization of DA neurons and 27 other cell types within their spatial context, identifying markers of healthy and aging cells [262].

In the case of neurodegenerative diseases such as PD and HD, significant strides have been made to correct disease mutations using gene editing techniques. In particular, zinc finger proteins (ZFPs) and clustered regularly interspersed short palindromic repeats/caspase 9 (CRISPR/Cas9) can be used as repair mechanisms to correct the abnormal CAG triplet expansion in HD [263]. The same system has been used to correct gene mutations, e.g., LRKK2, in preclinical models of PD [264,265]. In addition, phase I/II clinical trials using iPSC-derived DA cells have demonstrated feasibility, cell survival, DA production, and no tumor formation [266]. Striatal neuronal loss can be corrected by use of induced pluripotent stem cells (iPSCs). In our lab, we demonstrated improvement of the HD phenotype by implantation of human-embryonic-stem-cell-derived neural stem cells (hNSCs) in the striatum of genetic mouse models of HD [267]. These cells survive for long periods of time, integrate in host striatum, differentiate into MSNs and interneurons, and make synaptic connections with other MSNs [268]. Derived from these studies, a phase I/II clinical trial led by Dr. Leslie M. Thompson (University of California Irvine) is evaluating the safety of hNSCs implanted in HD patients.

In spite of undeniable progress made in the past decades to understand DA modulation, questions still remain. One is to try to understand DA modulation of large neuronal ensembles via volume transmission [269]. Similar to other neurotransmitters (e.g., GABA), there is a DA tone in several brain regions, suggesting that DA modulation not only occurs via synaptic communication and receptor binding. Volume transmission, whereby DA drifts away from the synapse, is known to occur in cerebral cortex and striatum [116,270]. This may allow for non-synaptic modulation of corticostriatal glutamate synapses through DA varicosities, as shown by use of cryo-correlative light and electron microscopy [271]. While, in the striatum, DA transporters (DATs) quickly recapture liberated DA, in other areas such as the frontal cortex, recapture is much slower due to the scarcity of the DA transporter (DAT) and has to rely on other transporters [272], facilitating widespread modulation of cortical neuronal ensembles. Another standing question is to understand the role of glia in DA modulation at the synaptic and extrasynaptic levels. Indeed, astrocytes are essential to regulate synaptic transmission in the brain [273,274]. Both astrocytes and microglia express DA receptors and help maintain DA homeostasis by continuous crosstalk [275]. More studies on the role of glial cells in DA–glutamate receptor interactions could lead to a better understanding of these interactions. Finally, as mentioned throughout this paper, the GluN2B subunit of NMDA receptors appears to play a critical role in numerous neurological and psychiatric conditions. In particular, GluN2B subunits at extrasynaptic locations underlie excitotoxicity [276,277]. When associated with activation of D1 receptors, toxic effects are exacerbated. Finding ways to perturb this association may prove therapeutic in many pathological conditions.

## 8. Conclusions

The study of DA and DA–glutamate receptor interactions continues to fascinate neuroscientists around the globe. Much has been learned in the past 60 years about their multiple effects and involvement in a wide array of normal functions and their role in a large number of pathological conditions. Studies of DA and its interactions with glutamate receptors have proven to be fertile ground. I can predict that future investigations will give more surprises and open new avenues for the treatment of an expanding number of neurological and psychiatric diseases.

## Data Availability

No new data were created or analyzed in this study. Data sharing is not applicable to this article.

## Supplementary Materials

The following supporting information can be downloaded at: https://www.mdpi.com/article/10.3390/cells15181627/s1; Figure S1: (A) The schematic shows the typical setup (used around the 1960’s-1980’s) for combined extracellular/intracellular unit recordings and microiontophoresis. In the case of cats, they were usually anesthetized with a short-acting barbiturate and placed in a stereotaxic frame. Anesthesia was maintained by continuous administration of 2% halothane, nitrous oxide, and O_2_. Craniotomy and dural retraction permitted access to stimulation and recording sites. Recording electrodes were glass micropipettes filled with K-citrate. Stimulation electrodes were implanted in the frontal cortex. In the case of rats, they were anesthetized with intraperitoneal injections of urethane and placed in a standard stereotaxic head-holder. Voltage fluctuations, extracellular or intracellular recordings were obtained from K-acetate-filled micropipettes with impedances between 30 and 60 MΩ. For iontophoresis, a five-barreled pipette was introduced in the caudate nucleus. One barrel served as a sharp recording electrode. Pipettes contained Glu, DA, GABA, NMDA, and saline for current balancing and control. Controls consist of application of positive or negative currents through the saline-containing pipette. (B) Setup for whole-cell patch recordings from CPNs or MSNs (used form the 1980’s to date). An upright microscope equipped with infrared (IR) light and Nomarski optics, and an IR sensitive camera were used to visualize individual neurons in the slices (~300 µm). Slices were maintained with oxygenated artificial cerebrospinal fluid (ACSF) and kept from moving with a platinum wire. Glass patch electrodes (3–5 MΩ) were filled with Cs-methanesulfonate for voltage clamp or K-Gluconate for current clamp recordings. Signals were amplified and digitized using pClamp or other software. (C) Imaging Glu release from corticostriatal terminals with FM1-43. (A) Cartoon of the basic striatal microcircuit. Glu, glutamatergic afferent; SN, substantia nigra; MSN, medium-sized spiny neuron; DA afferent; AMPA receptor; D, DA receptor subtype (D1 or D2). (B) A corticostriatal slice stained with 3,3′-diaminobenzidine shows the areas of stimulation and recording. Corticostriatal terminals from the primary motor cortex (MI) were loaded with FM1-43 by stimulation with bipolar electrodes placed over cortical layers V-VI. 2-photon microscopy images of corticostriatal terminals were obtained from the corresponding motor striatum). DA was released by local striatal stimulation. CC, corpus callosum (asterisk); AC, anterior commissure (diamond). Scale bar equals 1 mm. (C) Protocol for loading and destaining corticostriatal terminals with FM1-43. (D) (A) Stimulation of the forelimb motor cortex in the presence of FM1-43 resulted in labeled puncta in en passant arrays. Re-stimulation of the forelimb motor cortex at 10 Hz results in activity-dependent destaining. Stimulation began at t = 0. Scale bar equals 2 μm. (B) Corticostriatal terminal destaining is frequency dependent, showing differential response to stimulation at 1 Hz, 10 Hz, 20 Hz, and 50 Hz. Controls receiving either no stimulation or only striatal stimulation at 0.1 Hz show minimal destaining. Data points represent mean ± SEM. (C) The half-time decay of fluorescence intensity (t1/2) for 1, 10, 20, and 50 Hz stimuli shows the dependence of destaining on stimulation frequency.

## Appendix A. DA Modulation of Striatal or Cortical Excitability, Neurotransmitter Release, and Postsynaptic Glutamate Responses

**Effects of DA on MSN Excitability**			
**Preparation**	**Main Effect**	**Method(s)**	**References**
In vivo	Inhibition	Microiontophoresis	[2,3,4,5,6,7,8]
	Excitation/Inhibition	Electrical Stimulation of SNc	[9,10,11,13]
	Depolarization/AP block	Microiontophoresis	[14,15,16]
	Excitation (low dose, D1)	Microiontophoresis	[17,18,66]
	Inhibition (high dose, D1)		
In vitro	Excitation (low dose, D2)	Bath application	[21,22]
	Inhibition (high dose, D1)		
	Inhibition (D1)	Bath application	[20]
	Excitation (D1) RMP = −80 mV	Bath application	[23,24]
	Inhibition (D1) RMP = −60 mV		
**Effects on neurotransmitter release**			
Pharmacology	Inhibition (D2R)	Radioactive Glu, spectrometry	[42,44,45,46,47]
		Push–pull cannula, microdialysis	
In vitro/In vivo	Inhibition (D2R)	Iontophoresis; bath application	[19,48,49,50,54,56,58,60,62]
		2-PLSM (FM1-43)	
**DA-GluR receptor interactions**			
In vitro	Potentiation NMDA (D1)	Iontophoresis; bath application	[63,68,69,70,72,73,74,75,76,78,88,95,96,99]
	Attenuation NMDA/AMPA (D2)		
