# Supplementary material for: Multiple Mechanisms of Dopamine Modulation of Neuronal Excitability and Neurotransmission in the Striatum: A Personal and Historical Perspective
Source: Cells. 2026 Sep 8;15(18):1627. doi: 10.3390/cells15181627 (PMC13605292; doi:10.3390/cells15181627)

Methodology

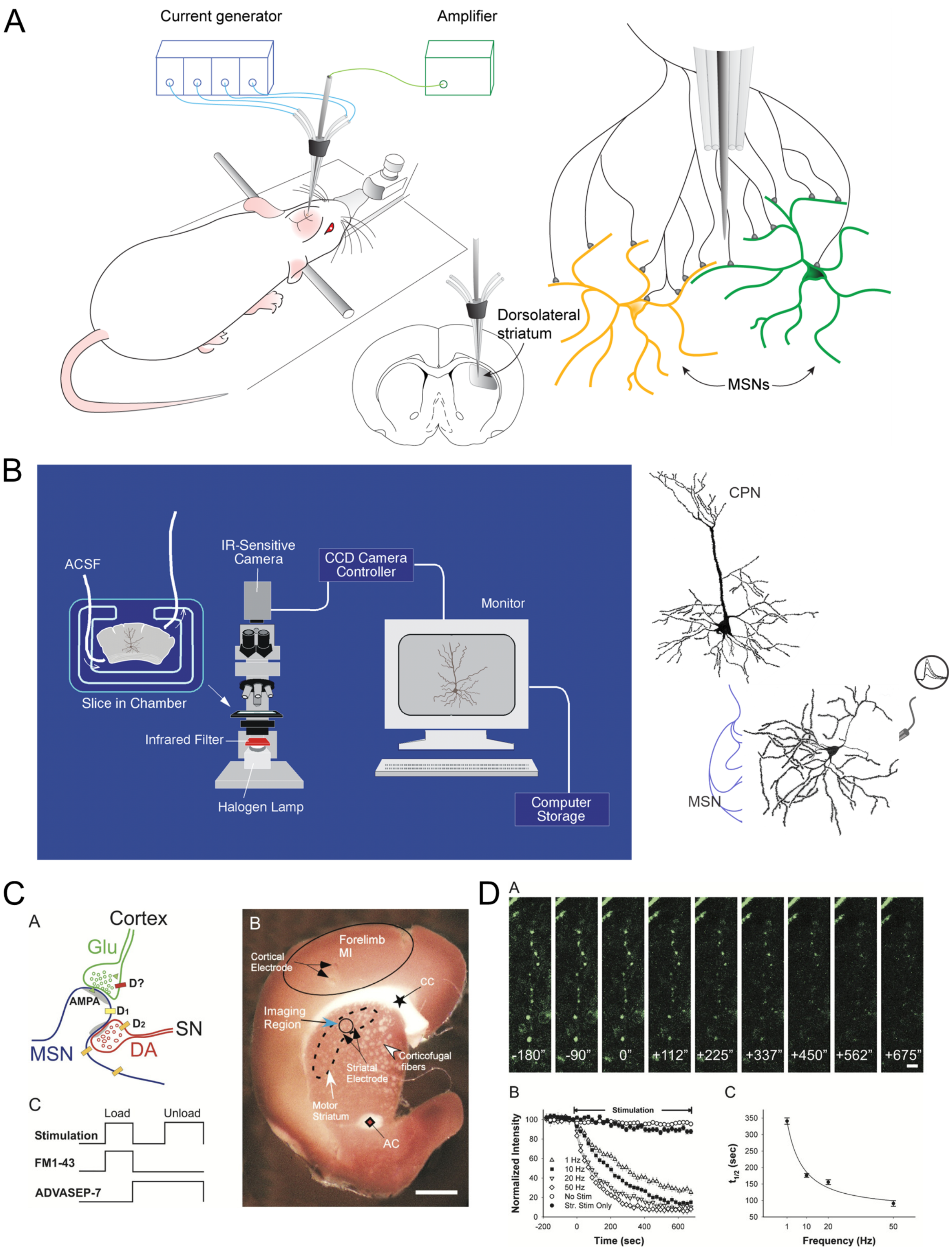

Supplement: Supplementary file 1 [file cells-15-01627-s001.zip › cells-4437527-supplementary figure S1.pdf]
